# Use of GetCheckedOnline, a Comprehensive Web-based Testing Service for Sexually Transmitted and Blood-Borne Infections

**DOI:** 10.2196/jmir.7097

**Published:** 2017-03-20

**Authors:** Mark Gilbert, Travis Salway, Devon Haag, Christopher K Fairley, Jason Wong, Troy Grennan, Zhaida Uddin, Christopher S Buchner, Tom Wong, Mel Krajden, Mark Tyndall, Jean Shoveller, Gina Ogilvie

**Affiliations:** ^1^ BC Centre for Disease Control Vancouver, BC Canada; ^2^ School of Population and Public Health University of British Columbia Vancouver, BC Canada; ^3^ Melbourne Sexual Health Centre Melbourne Australia; ^4^ Central Clinical School Monash University Melbourne Australia; ^5^ Division of Infectious Diseases Faculty of Medicine University of British Columbia Vancouver, BC Canada; ^6^ Ottawa Public Health Ottawa, ON Canada; ^7^ Fraser Health Vancouver, BC Canada; ^8^ Health Canada Ottawa, ON Canada; ^9^ Department of Pathology and Laboratory Medicine Faculty of Medicine University of British Columbia Vancouver, BC Canada; ^10^ British Columbia Centre for Excellence in HIV/AIDS Vancouver, BC Canada

**Keywords:** Internet, sexually transmitted diseases, diagnostic tests, health care delivery, health services research, intervention study

## Abstract

**Background:**

The British Columbia Centre for Disease Control implemented a comprehensive Web-based testing service GetCheckedOnline (GCO) in September 2014 in Vancouver, Canada. GCO’s objectives are to increase testing for sexually transmitted and blood-borne infections (STBBIs), reach high-prevalence populations facing testing barriers, and increase clinical STI service capacity. GCO was promoted through email invitations to provincial STI clinic clients, access codes to clients unable to access immediate clinic-based testing (deferred testers), and a campaign to gay, bisexual, and other men who have sex with men (MSM).

**Objective:**

The objective of the study was to report on characteristics of GCO users, use and test outcomes (overall and by promotional strategy) during this pilot phase.

**Methods:**

We used GCO program data, website metrics, and provincial STI clinic records to describe temporal trends, progression through the service pathway, and demographic, risk, and testing outcomes for individuals creating GCO accounts during the first 15 months of implementation.

**Results:**

Of 868 clients creating accounts, 318 (36.6%) submitted specimens, of whom 96 (30.2%) tested more than once and 10 (3.1%) had a positive STI diagnosis. The proportion of clients submitting specimens increased steadily over the course of the pilot phase following introduction of deferred tester codes. Clients were diverse with respect to age, gender, and ethnicity, although youth and individuals of nonwhite ethnicity were underrepresented. Of the 506 clients completing risk assessments, 215 (42.5%) were MSM, 89 (17.6%) were symptomatic, 47 (9.3%) were STI contacts, 232 (45.8%) reported condomless sex, 146 (28.9%) reported ≥4 partners in the past 3 months, and 76 (15.0%) reported a recent STI. A total of 63 (12.5%) GCO clients were testing for the first time. For 868 accounts created, 337 (38.8%) were by clinic invitations (0 diagnoses), 298 (34.3%) were by deferred testers (6 diagnoses), 194 (22.4%) were by promotional campaign (3 diagnoses), and 39 (4.5%) were by other means (1 diagnosis).

**Conclusions:**

Our evaluation suggests that GCO is an acceptable and feasible approach to engage individuals in testing. Use by first-time testers, repeated use, and STI diagnosis of individuals unable to access immediate clinic-based testing suggest GCO may facilitate uptake of STBBI testing and earlier diagnosis. Use by MSM and individuals reporting sexual risk suggests GCO may reach populations with a higher risk of STI. Motivation to test (eg, unable to access clinical services immediately) appears a key factor underlying GCO use. These findings identify areas for refinement of the testing model, further promotion, and future research (including understanding reasons for drop-off through the service pathway and more comprehensive evaluation of effectiveness). Increased uptake and diagnosis corresponding with expansion of the service within British Columbia will permit future evaluation of this service across varying populations and settings.

## Introduction

Globally, health systems are implementing new digital applications of existing health interventions to improve health care access and health outcomes. In the field of sexual health, Web-based testing services are widely considered to overcome barriers faced by individuals seeking testing for sexually transmitted and blood-borne infections (STBBIs) [1]. By offering an opportunity to test at a local laboratory or at home without needing to see a provider or present to a clinic, these services may eliminate known barriers related to conventional testing services (although to date this has not been well studied). Such barriers include feeling ashamed or embarrassed about getting a sexually transmitted infection (STI) test, fears of negative reactions from providers at disclosure of sexual behaviors, or clinic access barriers such as limited working hours or long wait times for appointments [2,3]. Web-based testing services are highly acceptable across all ages, offer privacy and anonymity, reach individuals at higher risk of STI, and may be cost-effective [4-7]. Web-based testing models are varied and are often designed as population screening programs for STIs, usually chlamydia [8,9]. Alternatively, Web-based testing can be offered as an integrated extension of existing clinical STI services and offer testing for multiple STBBIs, although fewer such models are known to exist or have been well evaluated in the published literature [5,10,11].

Following extensive consultation, formative research, and usability testing, the British Columbia Centre for Disease Control (BCCDC) implemented a comprehensive Web-based testing service for chlamydia, gonorrhea, syphilis, human immunodeficiency virus (HIV), and hepatitis C virus (HCV), which is operated as an integrated extension of its provincial STI clinic [12]. Called *GetCheckedOnline* (GCO) [13], the service has three main objectives: (1) to improve sexual health by increasing the uptake and frequency of STBBI testing and earlier diagnosis; (2) to reach populations with a greater prevalence of infection and facing barriers to testing access, such as gay, bisexual, and other men who have sex with men (MSM), youth, and people living in rural areas; and (3) to increase the capacity of STI clinic services and allow clinical resources to be more focused on more complex STI cases (eg, through reducing wait times and asymptomatic client visits).

In this study, our primary objective was to describe the use, test outcomes, and characteristics of GCO users during the first 15 months of operation. This pilot phase involved specimen collection sites in Vancouver, British Columbia, and focused on promotion to MSM and STI clinic clients in Vancouver. As a secondary objective, we aimed to describe differences in use and test outcomes between the strategies used to promote GCO.

## Methods

### Service Overview

The pilot phase of GCO began in September 2014. Details of the development process and service pathway have been described in detail elsewhere [12]. In brief, use of GCO involves proceeding through 5 steps: (1) account creation, (2) start and complete a risk assessment, (3) create and print a laboratory form, (4) submit specimens, and (5) receive results.

In step 1, clients create an online account, which includes collection of basic demographic information (eg, sex, age).

In step 2, clients answer questions on sexual history (eg, partner gender, prior testing history), which are collected in order to tailor test recommendations, educational messages, and testing reminders. Clients reporting symptoms or contact with a sexual partner with a diagnosed STI are recommended to go to a clinic to receive treatment but are not barred from proceeding with testing.

In step 3, clients view test recommendations, provide consent to get tested, and print their laboratory form.

In step 4, clients then present with the laboratory form to 1 of 6 designated collection centers in Vancouver where specimens are collected.

Finally, in step 5, clients receive a notification email when results are ready with a link to the GCO website to access their results. Results are provided online if all results are negative or by phone if any result is positive. If a test is invalid, such as a problem with a specimen, clients are notified to contact the clinic.

Steps 2 to 5 above constitute a single “test episode” and repeat each time a client uses GCO. Test recommendations are consistent with standard clinical practices of the BCCDC provincial STI clinic. Chlamydia (urine), gonorrhea (urine), HIV, and syphilis tests are recommended for all clients; HCV testing is recommended for clients reporting sharing drug paraphernalia and is available on an opt-in basis for MSM.

### Promotional Strategies

Access to GCO is available through email invitation or through access codes that can be entered on the GCO home page. Between September 9, 2014, and December 31, 2015, users were offered GCO through 1 of 3 promotional strategies: (1) email invitations to clients of the provincial STI clinic at BCCDC (hereafter referred to as “clinic client invitations”) beginning in September 2014; (2) access codes given to clients who presented for testing to the provincial STI clinic or 2 Vancouver STI clinics accessed by MSM but were unable to get a same-day appointment, or called for an appointment and did not want to wait (“deferred testers”), beginning in March 2015; and (3) access codes distributed through a promotional campaign emphasizing the convenience of the service to gay and bisexual men in Vancouver (“promotional campaign”) [14] between April and September 2015. During the latter half of the pilot phase, as interest in the service grew, access codes were also distributed by local STI clinics and gay men’s health organizations as well as to individuals contacting the BCCDC to request access (“other”).

### Data Collection and Analysis

Client demographics, risk assessment responses, and data on progression through each step in the service pathway were extracted from the GCO database. Specimen submission was considered our ultimate indication of service uptake, with the proportion of clients creating accounts proceeding to submit specimens at least once our primary measure of service uptake. STI diagnoses and relevant clinical and public health outcomes for GCO clients with a positive test result were extracted through a chart review of the electronic medical chart used by the provincial STI clinic for documenting all follow-up of positive STI diagnoses in Vancouver (including for GCO clients). Outcomes examined were result delivery (clinic staff contacted and provided clients with their positive result), treatment (client was treated using appropriate antibiotic regimens), and partner notification (documentation of whether sexual partners were notified or not).

For this analysis, we restricted data to clients who created accounts between September 9, 2014, and December 31, 2015. We included test episode outcomes through March 31, 2016, to allow adequate opportunity for those clients who created accounts late in the pilot enrollment period to order tests, submit specimens, and receive results. We examined progression through the 5 steps in the service pathway and described the demographic characteristics of GCO clients, responses to the risk assessment, repeated testing patterns, and customization patterns (opting in or out of recommended tests). We examined temporal trends by month for the number of accounts created, laboratory forms created, specimens submitted (counted once per test episode), and positive test results (excluding laboratory forms subsequently cancelled by clients, as well as repeated tests ordered <14 days after the initial test episode, which were assumed to be retests and part of the same testing episode). We also calculated 3-month moving averages for the percentage of clients who submitted specimens at least once. Finally, we stratified temporal trends and test episode outcomes by promotional strategy, using numbers of unique clients.

We used chi-square or *t* tests as appropriate (two-sided; *P*<.05 considered statistically significant). Analyses were completed using R version 3.1 (R Foundation for Statistical Computing). Institutional ethical review was not required, as this analysis constitutes an evaluation of a public health program and use of GCO program data in this way is permitted under its terms of use agreed to by all clients.

## Results

### Use of GetCheckedOnline and Testing Outcomes

**Figure 1:**
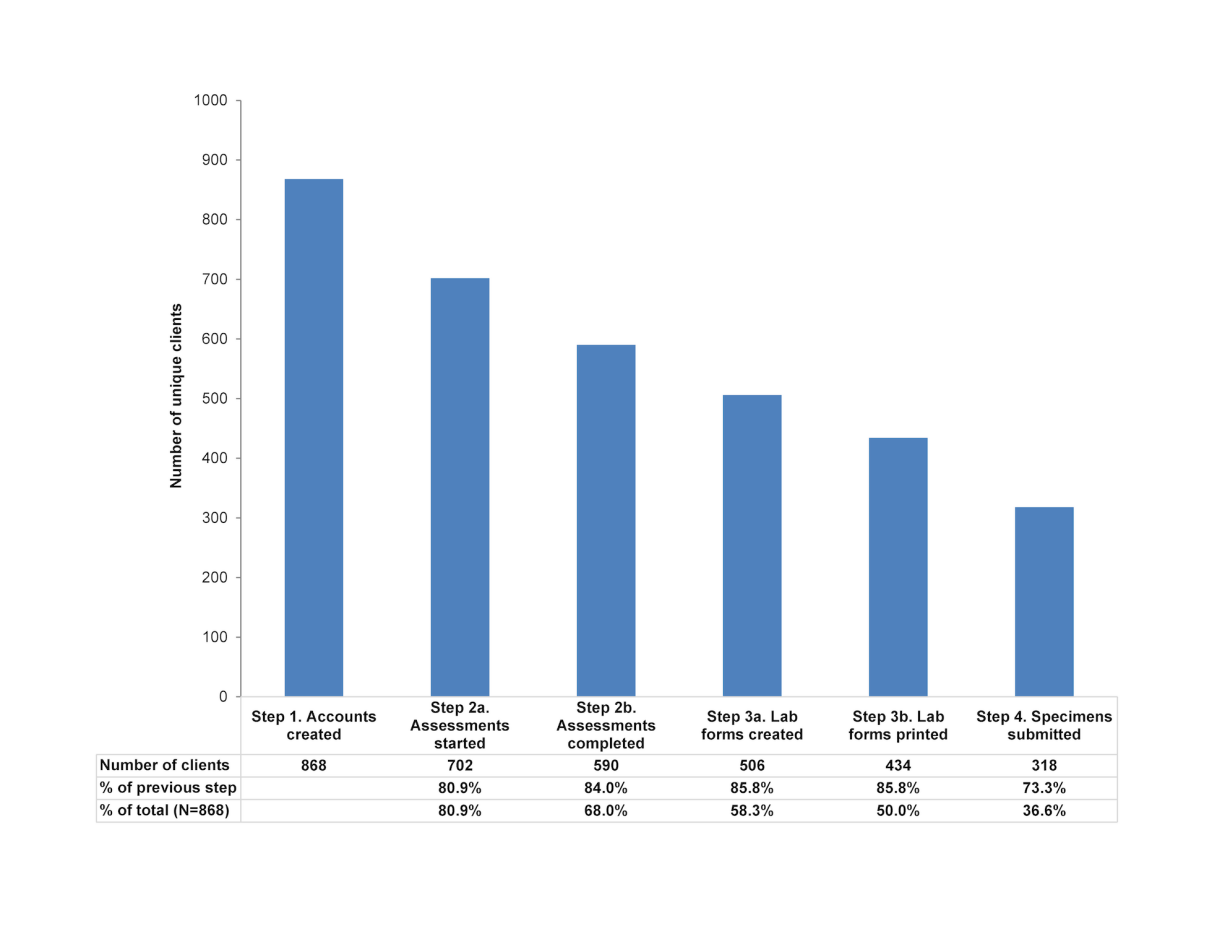
Completion of test episodes, by GetCheckedOnline service pathway steps.

Between September 2014 and December 2015, a total of 868 unique clients created GCO accounts. Approximately 15%-25% of clients discontinued at each step of the GCO testing process, with a cumulative attrition between account creation and specimen submission of 63.4% (Figure 1). A total of 30.8% of clients submitted specimens more than once (96/318, range 2-6 times) for a total of 462 submitted sets of specimens and an average interval between test episodes of 119 days (range 17-517 days). Among the 462 submitting specimens, 8 (1.7%) opted out of urine chlamydia and gonorrhea tests, 27 (5.8%) opted out of HIV tests, and 23 (5.0%) opted out of syphilis tests. Among the 34 completed episodes in which drug equipment sharing was reported, 3 (8.8%) opted out of HCV tests; among 175 completed episodes among MSM, 80 (45.7%) opted in to receive an HCV test.

Of the 318 clients submitting specimens, STI was diagnosed in 10 (3.1%) clients (3 with chlamydia only, 4 with gonorrhea only, 1 with chlamydia and gonorrhea dual infection, and 2 with syphilis only). Notably, a chlamydia and a gonorrhea infection were detected separately on 2 rectal swabs collected after self-collected swabs were introduced in February 2016, just before the end of the analysis period for test episode outcomes (March 31, 2016). Of these 10 clients, 2 had never before tested for STBBI, 4 reported symptoms of STI, and 1 reported contact with a partner with an STI. All clients received their results by phone within 6 days of diagnosis. A total of 5 clients were treated at the provincial STI clinic at BCCDC, 4 clients reported receiving treatment at another clinic, and 1 client did not confirm receipt of treatment. All 10 clients reported notifying partners.

For the 308 clients with negative results, 234 (76.0%) were known to have clicked the link in their notification email to access their test results. It is possible, however, that some clients logged in directly to their GCO accounts to access their results without using the notification link.

### Characteristics of Users

GCO clients were diverse with respect to age, gender, and ethnicity (Table 1). Clients ranged in age from 16 to 79 years, and 71.3% (619/868) identified as male. Reporting white (73.9 %, 566/766) and Chinese (10.2%, 78/766 ethnicity was most common; 1.7% (13/766) identified as First Nations or Métis. Most clients resided within Vancouver and surrounding suburbs.

**Table 1:** Characteristics of *GetCheckedOnline* clients provided during account creation, September 2014 to December 2015.

Characteristic^a^		Total accounts created (N=868)
Age in years, minimum-maximum (median)		16-79 (32)
**Age categories, years, n (%)**		
	16-19	9 (1.0)
	20-24	105 (12.1)
	25-29	217 (25.0)
	30-39	291 (33.5)
	40-59	215 (24.8)
	60+	31 (3.6)
**Gender, n (%)**		
	Male	619 (71.3)
	Female	240 (27.6)
	Transgender females (MTF^b^)	1 (0.1)
	Transgender males (FTM^c^)	5 (0.6)
	Other	3 (0.3)
**Ethnicity, n (%)**		
	First Nations^d^	10 (1.3)
	Métis	3 (0.4)
	White	566 (73.9)
	Chinese	78 (10.2)
	South Asian	20 (2.6)
	Filipino	15 (2.0)
	Korean	3 (0.4)
	Southeast Asian	9 (1.2)
	Japanese	3 (3.9)
	West Asian	5 (0.7)
	Latin American	15 (2.0)
	Black	6 (0.8)
	Arab	3 (0.4)
	Other	30 (3.9)
	No response^e^	102
**Region, n (%)**		
	City of Vancouver	534 (69.6)
	Suburban, Greater Vancouver	203 (26.5)
	Other	30 (3.9)
	No response or invalid entry^d^	101

^a^Restricted to unique *GetCheckedOnline* clients (ie, not counting multiple test episodes).

^b^MTF: male to female.

^c^FTM: female to male.

^d^No client identified as Inuit.

^e^Excluded from calculation of column percentages.

Among the 506 unique clients completing risk assessments, based on documentation on at least one risk assessment, 215 (42.5%) were males or transgender males who reported having male sex partners (MSM), 89 (17.6%) reported symptoms, 146 (28.9%) reported 4 or more sex partners in the past 3 months, 47 (9.3%) reported being a contact to an STI, 232 (45.8%) reported condomless anal or vaginal sex in the past 3 months (20/506, 4.0%, with an HIV-positive partner), 76 (15.0%) reported a recent STI diagnosis, and 41 (8.1%) had ever shared syringes or other drug paraphernalia. Of the 506 unique clients, 63 (12.5%) reported no previous test for STI or HIV at the time of completion of the first risk assessment; for 157 (31.0%) clients it had been more than 1 year since their last test. Responses indicating a potential need for HIV postexposure prophylaxis (based on condomless sex with an HIV-positive partner in the last 72 hours) or emergency contraception (based on condomless vaginal sex without other forms of birth control in the last 5 days) were provided at least once to 13 (2.6%) and 57 (11.3%) clients, respectively. The frequencies of risk assessment variables analyzed across all 695 risk assessments completed by these 506 clients are provided in Table 2.

**Table 2:** Responses to clinical assessment among *GetCheckedOnline* clients creating laboratory forms, N=695 assessments (completed by 506 clients).

Variable	Response categories	Per assessment (N=695)	
		n	%
Any symptoms reported^a^	Yes	103	14.8
	No	528	76.0
	Don’t know	40	5.8
	Prefer not to answer	24	3.5
Contact to an STI^b,c^	Yes	50	7.2
	No	458	65.9
	Don’t know	167	24.0
	Prefer not to answer	15	2.2
	Not applicable^d^	5	0.7
Gender of sex partners^e^	Males or transgender males with male partners (MSM^f^)	295	42.4
	Males or transgender males with female partners	255	36.7
	Males or transgender males with transgender partners	7	1.3
	Females or transgender females with male partners	159	22.9
	Females or transgender females with female partners	31	4.5
	Females or transgender females with transgender partners	2	1.2
	Other	1	0.1
	Prefer not to answer	18	2.6
Number of sex partners (vaginal, oral, or anal sex), last 3 months	0	22	3.2
	1	162	23.3
	2-3	294	42.3
	4-9	158	22.7
	10+	30	4.3
	Don't know	1	0.1
	Prefer not to answer	27	3.9
	Not applicable	1	0.1
Types of sex, last 3 months^e^	Vaginal	406	58.4
	Oral receiver	587	84.5
	Oral giver	537	77.3
	Anal bottom	188	27.1
	Anal top	223	32.1
	Prefer not to answer	26	3.7
Condomless anal or vaginal sex, last 3 months	No	372	53.5
	Yes	286	41.2
	Don't know	9	1.3
	Prefer not to answer	25	3.6
	Not applicable	3	0.4
Condomless anal or vaginal sex with HIV^g^-positive partner, last 3 months	No	450	64.7
	Yes	22	3.2
	Don't know	157	22.6
	Prefer not to answer	18	2.6
	Not applicable	48	6.9
Condomless (or condom broke during) anal or vaginal sex with partner known or thought to be HIV-positive, last 72 hours^h^	No	573	82.4
	Yes	14	2.0
	Don't know	44	6.3
	Prefer not to answer	17	2.4
	Not applicable	47	6.8
Condomless (or condom broke during) vaginal sex without using other form of birth control, last 5 days^i^	No	485	69.8
	Yes	63	9.1
	Don't know	12	1.7
	Prefer not to answer	19	2.7
	Not applicable	116	16.7
STI diagnosis, last 12 months	No	561	80.7
	Yes	103	14.8
	Don't know	15	2.2
	Prefer not to answer	16	2.3
Shared drug equipment	No	606	87.2
	Yes	57	8.2
	Don't know	5	0.7
	Prefer not to answer	13	1.9
	Not applicable	14	2.0
Last STI or HIV test	Never	64	9.2
	Last 3 months	162	23.3
	3-6 months	170	24.5
	6 months to 1 year	117	16.8
	>1 year	157	22.6
	Don't know	12	1.7
	Prefer not to answer	14	2.0

^a^Symptoms listed: painful urination, sores on or near genitals, rash on any part of the body, anal discharge, pain, blood, lesion, vaginal discharge, odor, itch, abnormal vaginal bleeding, lower abdominal pain, pain during intercourse, discharge from penis, swelling in testicles.

^b^STI: sexually transmitted infection.

^c^Sex partner who has recently tested positive for STI or told respondent he or she needs to get tested.

^d^Not applicable: client selected response.

^e^Categories not mutually exclusive, thus numbers do not sum to 100%.

^f^MSM: men who have sex with men.

^g^HIV: human immunodeficiency virus.

^h^Respondents who answered “yes” were shown information about how to access HIV postexposure prophylaxis.

^i^Respondents who answered “yes” were shown information about how to access emergency contraception.

### Temporal Trends and Differences by Promotional Strategy

**Figure 2:**
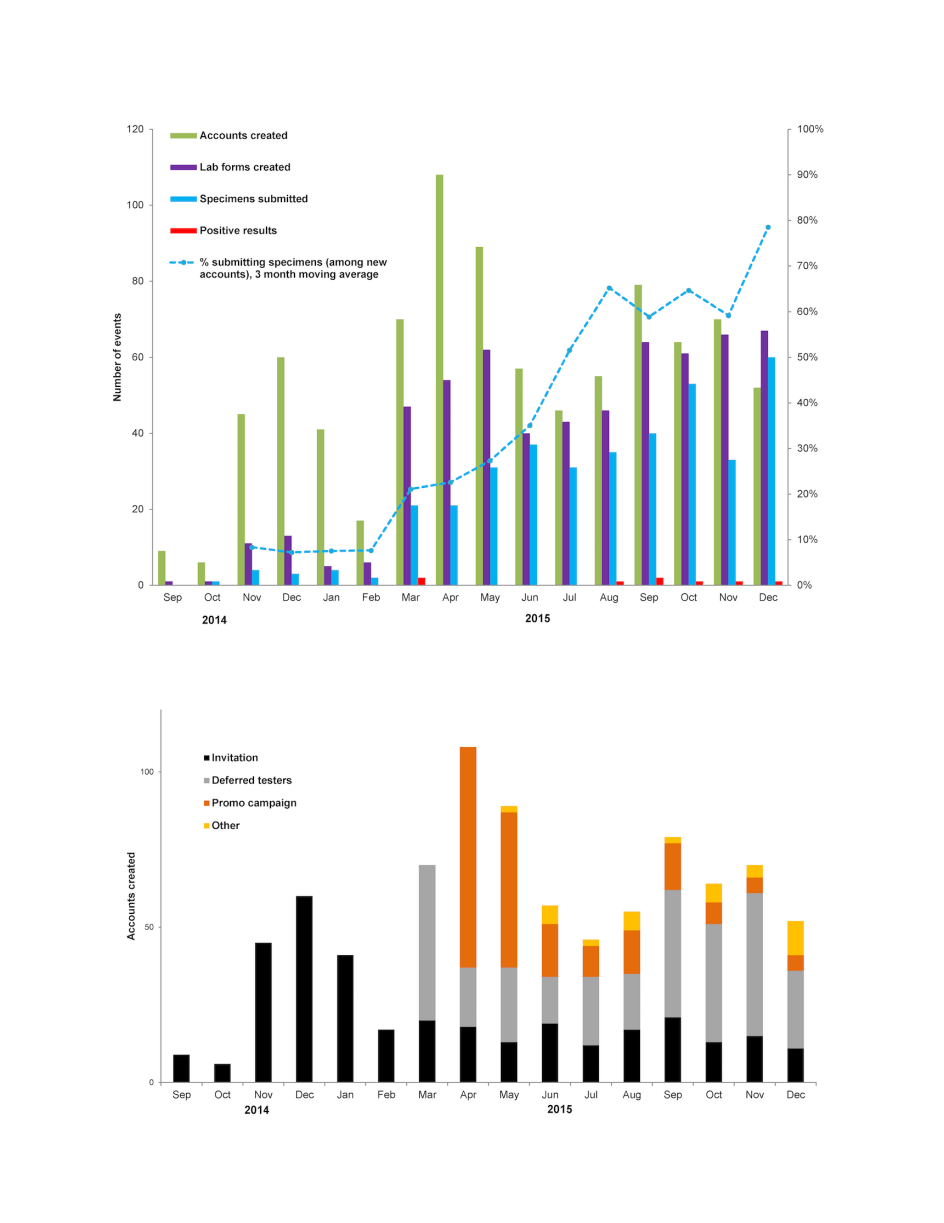
Promotion and uptake of GetCheckedOnline by month; (top) accounts, lab forms, specimen submissions, and positive results; (bottom) accounts created by promotional strategy.

Account creation per month increased from the GCO program launch in September 2014 to a peak in April 2015, coinciding with the launch of the deferred tester codes and the promotional campaign (Figure 2). The percentage of clients proceeding from account creation to at least one specimen submission increased from 8.3% in the first 3 months of the pilot phase when accounts were created by clinic client invitations to 78.5% at the end of the pilot phase (*P*<.001) when the majority of accounts were created by deferred testers.

We observed large and statistically significant differences in progression through the steps of the service when examined across the 3 promotional strategies (Table 3). Of 868 accounts, 337 (38.8%) were created by clinic invitees, 298 (34.3%) by deferred testers, and 194 (22.4%) by promotional campaign clients; 113/130 (86.9%) of campaign clients who created a laboratory form were MSM. The percentage of clients submitting specimens was highest for deferred testers (184/298, 61.7%), followed by promotional campaign clients (58/194, 29.9%) and clinic invitees (62/337, 18.4%; *P*<.001). Of the 10 clients with positive results, 6 were deferred testers, 3 were promotional campaign clients, and 1 was referred by a local gay health organization. Differences in percent positivity by promotional strategy were not significant, although statistical power was limited given the small number of positive results.

**Table 3:** Uptake of *GetCheckedOnline* and related steps of service pathway, by promotional strategy.

Promotional strategy^a^	Accounts created n (%)	Laboratory forms created^b,c^ n (%)	Specimens submitted^b,c^ n (%)	Positive results^d^ n (%)	Repeated testing^b,d^ n (%)
Clinic client invitations	337	102 (30.3)	62 (18.4)	0 (0.0)	29 (46.8)
Deferred testers	298	249 (83.6)	184 (61.7)	6 (3.3)	39 (21.2)
Promotional campaign	194	130 (67.0)	58 (29.9)	3 (5.2)	23 (39.7)
Other^e^	39	25 (64.1)	14 (35.9)	1 (7.1)	5 (35.7)
Total	868	506 (58.3)	318 (36.6)	10 (3.1)	96 (30.2)

^a^All metrics restricted to unique clients only (in order to calculate percentages with proper denominators).

^b^*P*<.05 for chi-square test comparing proportions across promotional strategies.

^c^Denominator is accounts created.

^d^Denominator is those who submitted specimens.

^e^Includes referrals from other primary care and community clinics, gay health organizations, and those requesting access.

## Discussion

### Principal Findings

In this evaluation, we have shown GCO to be an acceptable and feasible approach to engage clients in STI testing. These preliminary findings suggest that broader implementation of GCO may have the potential to improve sexual health and increase uptake and frequency of STBBI testing, given the repeated use by clients and use by both clients who had never previously tested for STI or HIV and clients reporting it had been more than 1 year since they were last tested. Of the 10 individuals with a positive result, 6 were deferred testers who were seeking testing but unable to be seen immediately in a clinic; it is possible that these individuals accessed testing and their STI was diagnosed sooner through GCO. Furthermore, the addition of self-collected throat and rectal swabs just before the end of the analysis period led to the diagnosis of STI in 2 clients, confirming the importance of including these testing options as part of a Web-based testing service to diagnose STI that may otherwise be missed [15]. Beyond testing, clients reporting specific risk events also received tailored educational messages regarding postexposure prophylaxis and emergency contraception.

GCO appears to also be reaching individuals who may be at greater risk of infection. Of GCO clients, 42% were MSM and, based on responses to the risk assessment, a large proportion of GCO clients reported risk factors for STBBI, including condomless sex (41%), prior STI (15%, in past 12 months), high number of partners (27%, 4+ in past 3 months), and sharing of syringes or drug paraphernalia (8%). We also hypothesize that GCO may be reaching individuals who face barriers to accessing clinic-based STBBI testing. For example, clients reported a range of stigmatized gender identities and same-gender sexual activities, who may be more likely to face testing barriers through conventional testing services (eg, discomfort disclosing sexual identity or sexual behavior because of associated stigma) [16,17]. However, some populations facing testing barriers to STBBI testing are not yet well-represented among GCO clients. Individuals younger than 25 years of age comprised 13% of GCO users yet, in 2014, comprised 50% and 28% of chlamydia and gonorrhea infections in British Columbia [18]. There are also inequities based on ethnicity; almost three-quarters of GCO clients reported white ethnicity, yet 42% of respondents in the 2006 census for the Greater Vancouver region identified as a visible minority [19]. Consideration of how GCO may be promoted or adapted for youth and diverse ethnicities is needed.

During this GCO pilot phase, the provincial STI clinic was able to offer testing to 318 additional clients, of which the majority were asymptomatic. While representing a small percentage of the total clients seen by the provincial STI clinic during this time period, these findings suggest that GCO has the potential to offset growing demands on clinic capacity and that this potential will increase over time as use of the service expands. However, despite recommendations to visit a clinic, a small number of clients with symptoms or who had a partner with an STI proceeded to test through GCO. As these clients may have not received appropriate clinical management, further research is needed to understand the motivations of these clients for using GCO and whether any program modifications are needed.

Overall, 3% of clients testing through GCO had a new STI diagnosis, which may be lower than typical of many STI clinic settings. If we consider diffusion of innovations theory, innovators and early adopters of GCO may not necessarily reflect the risk profile of the future population of GCO clients and so diagnosis rates may change over time [20]. Interestingly, we note as demonstrated in Figure 2 (top) that the majority of the STI diagnoses were made after July 2015, suggesting there may be temporal trends toward increasing risk of infection among GCO users that may be explained by this theory. We postulate that a lower prevalence of STBBIs among GCO users may also be a reflection of the intervention itself, as clients experiencing symptoms or having a partner with a diagnosed STI (and thereby a higher probability of infection) are recommended not to proceed and instead to present to a clinic for testing. We also had no users with diagnosed HIV or HCV, which may reflect lower overall prevalence of these viral infections compared with bacterial STIs or that awareness or uptake of GCO has not yet penetrated higher-risk sexual networks for these infections. With accumulation of more data we hope to be able to investigate these questions and examine more carefully how prevalence may differ across different subgroups of GCO users.

### Service Pathway

We observed consistent drop-off throughout the steps of the service pathway (Figure 1). This is not unexpected, having been reported for other Web-based STI testing services involving downloading laboratory requisitions (where between 10% and 33% of clients downloading requisitions submit specimens for testing) [11,21]. We postulate that many individuals who create an account and do not submit specimens are curious and learning about the service but not motivated to test at that time. However, the drop-off may represent true barriers posed by the GCO service itself (eg, need to print laboratory requisitions, dissatisfaction with the testing model). Client interviews and further analysis of GCO program data such as website metrics, time between steps, and characteristics of clients completing and not completing testing are being undertaken to determine if there may be modifiable factors related to website design or the GCO model that could facilitate progress through the service pathway (eg, optimizing the user experience, reminders of specimens not submitted).

### Effect of Promotional Strategies

Motivation to test appeared to increase over the pilot phase, with an increased proportion of individuals submitting specimens over time, which is likely explained by differences between promotional strategies. The first promotional strategy used was email invitations to clinic attendees, which had the lowest specimen submission rate and no resulting diagnoses; as emails were collected from STI clinic clients, many of whom had just completed a testing encounter, motivation to use GCO was likely low. Deferred testers were likely the most motivated to get tested through GCO as they were deliberately seeking testing at the time of learning about GCO; unsurprisingly, this group had the highest specimen submission rates. While specimens submitted by MSM had the highest positivity, only 58 specimens were submitted through the promotional campaign. As we have previously found high intentions to use GCO among Canadian MSM [22], we hypothesize that this may be related to the availability of existing low-barrier testing clinics for MSM in Vancouver, and the perceived benefits of seeing health care providers in these clinics who are competent in providing sexual health care for MSM are greater than the perceived benefits of GCO. Further evaluation of the campaign and reach of GCO among MSM in Vancouver is underway. Regardless, these findings suggest that clinic-based promotion (particularly for deferred testers) is an effective means of engaging individuals to use GCO.

### Limitations

While most of the data presented in this report are administrative records of service use, some analyses are based on self-reported data collected routinely through GCO. While we generally saw high levels of completion for the risk assessment questions, ethnicity and region were not reported for 12% of individuals creating accounts, which may affect our conclusions about these variables. Many outstanding questions remain about the individual, health service, and population impacts of GCO that cannot be answered using program data, including understanding the experiences of clients choosing to use GCO, how they compare with clinic clients in terms of risk of infection and testing barriers, and the impacts on testing patterns and prevention of ongoing transmission. We are currently undertaking a comprehensive program of research that will aim to answer these questions, as well as further confirm these preliminary, suggestive findings [12].

### Conclusions

A comprehensive, integrated STBBI testing service has been successfully launched in British Columbia. GCO is the first Web-based testing service to offer testing for multiple STBBIs in Canada, and our study adds to the sparse literature on the impact of online comprehensive STBBI testing as an extension of existing STI services. Our findings point to possible areas for refinement of the testing model and promotional strategies, such as optimizing the user experience, promoting to youth and visible minorities, and targeting individuals motivated to test. In February 2016, GCO was expanded to 2 other regions in British Columbia in partnership with regional health authorities, leading to a large increase in uptake and diagnoses. Ongoing evaluation of GCO will allow us to evaluate more fully whether our program objectives have been achieved, describe the implementation of this novel intervention across a range of populations and settings in British Columbia, and is critical for ensuring ongoing funding and sustainability of the program. The lessons learned from a comprehensive evaluation of GCO may also be more broadly relevant for understanding the impact of other self-care interventions and supporting diagnostic services for the public. Ultimately, services like GCO may prove a useful complement to—not replacement of—existing clinic or outreach-based services, as one intervention in a suite of necessary interventions needed to effectively meet the health care needs of a diverse population.

## Abbreviations

BCCDC: British Columbia Centre for Disease Control
GCO: GetCheckedOnline
HCV: hepatitis C virus
HIV: human immunodeficiency virus
MSM: men who have sex with men
STBBI: sexually transmitted and blood-borne infection
STI: sexually transmitted infection
